# Validity and reliability of total body volume and relative body fat mass from a 3-dimensional photonic body surface scanner

**DOI:** 10.1371/journal.pone.0180201

**Published:** 2017-07-03

**Authors:** Carolin Adler, Astrid Steinbrecher, Lina Jaeschke, Anja Mähler, Michael Boschmann, Stephanie Jeran, Tobias Pischon

**Affiliations:** 1 Molecular Epidemiology Research Group, Max Delbrück Center for Molecular Medicine, Berlin, Germany; 2 Experimental & Clinical Research Center—a joint cooperation between Charité-Universitätsmedizin Berlin and Max Delbrück Center for Molecular Medicine, Berlin, Germany; 3 Charité–Universitätsmedizin Berlin, Berlin, Germany; 4 DZHK (German Center for Cardiovascular Research), partner site Berlin, Berlin, Germany; Hospital Universitario de la Princesa, SPAIN

## Abstract

**Objective:**

Three-dimensional photonic body surface scanners (3DPS) feature a tool to estimate total body volume (BV) from 3D images of the human body, from which the relative body fat mass (%BF) can be calculated. However, information on validity and reliability of these measurements for application in epidemiological studies is limited.

**Methods:**

Validity was assessed among 32 participants (men, 50%) aged 20–58 years. BV and %BF were assessed using a 3DPS (VitusSmart XXL) and air displacement plethysmography (ADP) with a BOD POD^®^ device using equations by Siri and Brozek. Three scans were obtained per participant (standard, relaxed, exhaled scan). Validity was evaluated based on the agreement of 3DPS with ADP using Bland Altman plots, correlation analysis and Wilcoxon signed ranks test for paired samples. Reliability was investigated in a separate sample of 18 participants (men, 67%) aged 25–66 years using intraclass correlation coefficients (ICC) based on two repeated 3DPS measurements four weeks apart.

**Results:**

Mean BV and %BF were higher using 3DPS compared to ADP, (3DPS-ADP BV difference 1.1 ± 0.9 L, p<0.01; %BF difference 7.0 ± 5.6, p<0.01), yet the disagreement was not associated with gender, age or body mass index (BMI). Reliability was excellent for 3DPS BV (ICC, 0.998) and good for 3DPS %BF (ICC, 0.982). Results were similar for the standard scan and the relaxed scan but somewhat weaker for the exhaled scan.

**Conclusions:**

Although BV and %BF are higher than ADP measurements, our data indicate good validity and reliability for an application of 3DPS in epidemiological studies.

## Introduction

Obesity is a major risk factor for non-communicable diseases like type-2-diabetes mellitus and cardiovascular diseases, and accounts for a substantial proportion of disability-adjusted life years and mortality worldwide [1].

The definition of obesity is based on the body mass index (BMI) [2, 3]; however, although BMI is, to some extent, correlated with the amount of fat, it is neither a specific marker of body fat nor a marker for abnormal fat accumulation. For example, for the same BMI older adults tend to have a higher body fat percentage, and therefore health risk assessment using BMI is less accurate in individuals >65 years of age [4]. Similarly, for the same BMI the percentage of body fat is usually higher among Asian people compared to Western populations [5]. Yet, the assessment of body fat is challenging and most methods used today have potential drawbacks. Air- and water displacement techniques, which are most widely used, rely on rigid measurement conditions and even slight alterations in room temperature, humidity and pressure during the measurement can lead to invalid results [6]; further, participants should not have eaten or been physically active two hours prior to investigation. When using a bioelectrical impedance analysis for assessing the body composition, similarly, participants should be fasting for at least two hours and not be physically active for 12 hours [7]. Finally, dual energy X-ray absorptiometry computed tomography and magnetic resonance imaging are expensive and might constitute a risk for participants [8–11].

Three-dimensional photonic body surface scanners (3DPS) provide an opportunity for a standardized acquirement of data on total body volume (BV). This is promising particularly for large-scale epidemiological studies, where standardized, fast, accurate, and precise assessment methods are key issues for a valid estimation of disease risk. In contrast to the abovementioned methods to assess the body composition, the 3DPS examination does not require the participants to be in a fasting state or to restrict physical activity beforehand. In Germany, measurement of body size has been recently conducted for the clothing industry in more than 13,000 individuals using a laser-based 3DPS (VitusSmart XXL, Human Solutions GmbH, Kaiserslautern, Germany) [12]. Within 12 seconds, the 3DPS scans the body surface using four eye-safe lasers, produces a 3D image, and calculates 153 body size measures relevant for the clothing industry. We have recently shown that automated measurement of waist and hip circumference using this 3DPS is feasible and has good validity and excellent short-term reliability as compared to manual measurement according to WHO standards [13]. However, validity and reliability of measurement of total BV and body fat percentage (%BF) using 3DPS are unclear. Knowing these is important since in the context of epidemiologic studies one usually assesses risk factors like BV only once aiming to assess the ‘true’ exposure over longer time periods, which is then used to draw exposure-disease associations, i.e., to what extent persons with higher or lower BV differ with respect to chronic disease risk. Low within- or high between-person variance between two measures will result in a high reliability [14], which is a precondition to derive approximate true, unattenuated relative risks based on a single risk factor measurement in epidemiological studies [15].

The aim of this study was therefore to assess the validity of 3DPS total BV and %BF measures against air displacement plethysmography (ADP) measures and to identify factors potentially contributing to a measurement disagreement in the general adult population. Further, as we have already shown a good short-term reliability of the 3DPS [13], we examined the longer-term, and thus predominantly biological reliability of 3DPS total BV and %BF estimates over a period of approximately four weeks to evaluate applicability of the 3DPS in epidemiological studies.

## Materials and methods

### Study population

Participants were recruited from February 2016 to June 2016 as part of the larger-scaled MetSScan study, which targeted a sample size of 500 participants and was conducted by the Molecular Epidemiology Research Group of the Max Delbrück Center for Molecular Medicine in the Helmholtz Association, Berlin. The MetSScan study aims to develop algorithms for the assessment of parameters of the metabolic syndrome [16] using a 3DPS.

Participants for the main study were recruited based on institution's internal mailing lists, local press releases, newspaper articles, and advertisements as well as public postings. Inclusion criteria were age 18–79 years, sufficient German language skills and the ability to give informed consent. Exclusion criteria were current pregnancy or breastfeeding, known hemophilia, anticoagulant medication, dependency on medical appliances for stand and movement, or current wearing of a plaster cast.

For assessment of validity of total BV measurements, ADP measurements were performed on 32 participants in addition to the main study protocol. ADP measurements were not taken in persons with claustrophobia.

To assess longer-term reliability (i.e., biological reliability) of BV and %BF, the 3DPS measurements were repeated after approximately four weeks in an independent sample of 18 participants under identical conditions.

The study was approved by the Ethics Committee of the Charité—Universitätsmedizin Berlin and by the local data protection officer. Informed written consent was obtained from all participants.

### Measurements

All participants completed a study protocol at the study center comprising measurement of height and weight, the 3DPS and ADP examination. Due to the original aim of the MetSScan study, we needed a fasting blood sample to determine blood glucose. Thus participants were in a fasting state for at least 8 hours prior to the investigation. All examinations were taken on the same day with a maximum interval of one hour between the 3DPS and ADP measurements.

For all examinations, participants wore minimal, tight-fitting, unpadded underwear, no jewelry or eyeglasses and a bathing cap. The measurement of body height followed standardized procedures according to WHO guidelines [17] using a portable stadiometer (seca 285, SECA GmbH & Co. KG, Hamburg, Germany, precision: 0.1 cm).

The 3DPS examination was performed with a VitusSmart XXL and the software Anthroscan Professional version 3.3.0 (Human Solutions GmbH, Kaiserslautern, Germany). The measurement principle is based on optical triangulation, whereby the scanner uses eight eye-safe laser sensor heads and cameras to create a 3D point cloud of the human body surface. The measuring range covers 2100 mm height x 1000 mm width x 1200 mm depth. The accuracy is within a level of ±1 mm and the scanner reaches a density of 27 points/cm^2^ within a scan time of 10 seconds. Based on the 3D point cloud, 153 anthropometric measures are computed by the software according to international standard DIN EN ISO 20685 [18]. Further, the calculation of total BV is part of the software.

The study protocol for the 3DPS measurement consisted of three consecutive scans in three different postures: 1. standard position: upright posture with legs shoulder-wide apart and, if possible, no contact of thighs; arms slightly bend and away from the body; hands making a fist with thumbs showing forward; and head positioned compliant with the Frankfort horizontal plane (StdScan), 2. relaxed position: legs hip-wide apart, arms hanging relaxed without contact to the body, hands making a fist, head aligned in Frankfort horizontal plane (RlxScan), and 3. relaxed position after maximum expiration (ExhScan). Except for the ExhScan, participants were asked to breathe normally during the scan procedure. All scan images were immediately visually checked by the study personnel. A scan was repeated if the scan quality did not reach pre-defined standards, e.g. with regard to image artifacts, deficient resolution, or if the posture was not correct. The scan measurement series was cancelled in case the quality remained insufficient after three repeated attempts in the same individual. The 3DPS was calibrated prior to the first examination of each day with a cylinder tube of defined height and width provided by the manufacturer.

The assessment of BV by ADP was performed with a BOD POD^®^ (model number: BOD POD 2007A, COSMED USA, Inc., Concord, USA), which shows high short-term reliability [19–22], and followed the instructions given by the manufacturer’s software (version 5.4.1). Hence, the BOD POD^®^ was calibrated each day with a 50 l calibration cylinder, allowing for a maximum standard deviation (SD) of 50 ml in a series of five volume measurements. Body weight was measured with an integrated electronic scale (precision: ±10 g, maximum weight: 250 kg) connected to the BOD POD^®^, which was calibrated every 14 days with two pre-specified 10 kg weights. Body height was taken from the above described stadiometer measurement. Directly before each measurement, an obligate 2-step calibration was carried out: first, the volume of the chamber was measured without participant inside and then again with the 50 l calibration cylinder placed inside. After the participant entered the chamber, two measurements of raw BV were executed. If these two measurements differed by 150 ml or 0.2% of BV, a third measurement was performed and the two closest measurements within these agreement criteria were averaged. The estimate of raw BV was adjusted for thoracic gas volume (TGV) predicted from gender, age, and height by the software (8) and a body surface area artifact, which arises due to the apparent negative volume of air under isothermal conditions related to the skin surface [6]. Using the corrected BV (BV_corr_), body density was calculated as:

$$Body\;density=\frac{body\;mass\;\left(kg\right)}{BV_{corr}\left(L\right)}$$ (1)

which was subsequently used to calculate %BF with Siri’s equation in normal- and overweight individuals (BMI 18.5–30.0 kg/m^2^) [23] or with Brozek’s equation in very lean or obese individuals (BMI <18.5 or >30.0 kg/m^2^) [24]:

$$\text{Siri}:\;\%BF=\left(\frac{4.95}{\mathrm{body}\;\mathrm{density}}-4.50\right)\times 100$$ (2)

$$\text{Brozek}:\%BF=\left(\frac{4.57}{\mathrm{body}\;\mathrm{density}}-4.142\right)\times 100$$ (3)

All calculations were automatically executed by the BOD POD^®^ software.

The 3DPS derived BV was corrected for TGV for the StdScan and RlxScan, and residual volume (VR) for the ExhScan, respectively. TGV was calculated according to the following equations by Brozek et al. [24] and Crapo et al. [25] based on functional residual capacity (FRC) and tidal volume (VT).

TGV=FRC+0.5×VT (4)

Men:FRC=0.0472×height+0.0090×age−5.290;VT=1.2 (5A)

Women:FRC=0.0360×height+0.0031×age−3.182;VT=0.7 (5B)

VR was calculated with equations developed by Crapo et al. [18]:

Men:VR=0.0216×height+0.0207×age−2.840 (6A)

Women:VR=0.0197×height+0.0201×age−2.421 (6B)

%BF was calculated for all corrected BVs of 3DPS scans using Eqs (2) and (3).

### Statistical analysis

Data are presented as mean ± standard deviation (SD). BMI was calculated as body weight in kilogram (kg) divided by body height in meter squared (m^2^) and was used to define non-overweight (BMI < 25 kg/m^2^, including underweight), overweight (BMI≥25 to <30 kg/m^2^) and obesity (BMI≥30 kg/m^2^) [26]. The comparison of 3DPS and ADP was performed after correction of the 3DPS derived BV for TGV for the StdScan and RlxScan, or VR for the ExhScan, respectively. The differences (3DPS–ADP) in BV and %BF were calculated and stratified by gender, age, and BMI. The validity of 3DPS BV and %BF was assessed by analysis of agreement using Pearson correlation coefficients and Bland Altman plots of the difference between BV assessed by 3DPS and ADP, or %BF respectively against the respective mean [27, 28]. Q-Q plots were used to examine normal distribution of the 3DPS and ADP BV and the BV differences between 3DPS and ADP [29]. Since BV and the BV differences were not normally distributed, Wilcoxon signed ranks test for paired samples was used to test for the significance of measurement differences between both methods. Further, the sample was stratified by gender, age and BMI, the difference of BV or %BF between both methods was calculated and the mean difference of the stratified samples was tested for significance with the Mann–Whitney U test.

Multiple regression analyses were used to predict ADP BV based on 3DPS BV including gender, age, BMI, and an interaction term of 3DPS BV*gender. The final models retained only the significant factors after a stepwise exclusion procedure.

For the assessment of reliability, the differences in the duplicated 3DPS measurements (BV and %BF), calculated as scan at date 1 –scan at date 2, and the intraclass correlation coefficients (ICC) ± 95%CI were calculated.

P-values presented are 2-tailed and p<0.05 were considered statistically significant. The analyses were performed using SPSS 18 (IBM Corporation, Armonk, USA) and SAS Enterprise Guide 4.3. (SAS Institute Inc., Cary, USA).

## Results

No technical issues or refusal of participants occurred during the completion of the study and quality checks revealed formal plausibility of the data obtained. Basic characteristics of the two subsamples including 32 and 18 participants are summarized in Table 1. All participants in our study were of Caucasian origin.

**Table 1:** Characteristics of the validation sample and the reliability sample.

	validation sample		reliability sample	
	N	%	N	%
total	32	100	18	100
men	16	50	6	33.3
women	16	50	12	66.6
age				
18–29 years	7	21.9	4	22.2
30–49 years	19	59.4	11	61.1
50–79 years	6	18.8	3	16.7
BMI				
<25.0	18	56.3	11	61.1
25.0–29.9	11	34.4	5	27.8
> = 30.0	3	9.4	2	11.1
	median	interquartile range	median	interquartile range
height (cm)	172.0	10.8	168.6	14.3
weight (kg)	76.3	18.1	75.6	22.8
time interval between visits (weeks)	-	-	4.0	2.0

### Validation of 3DPS against ADP

**Fig 1:**
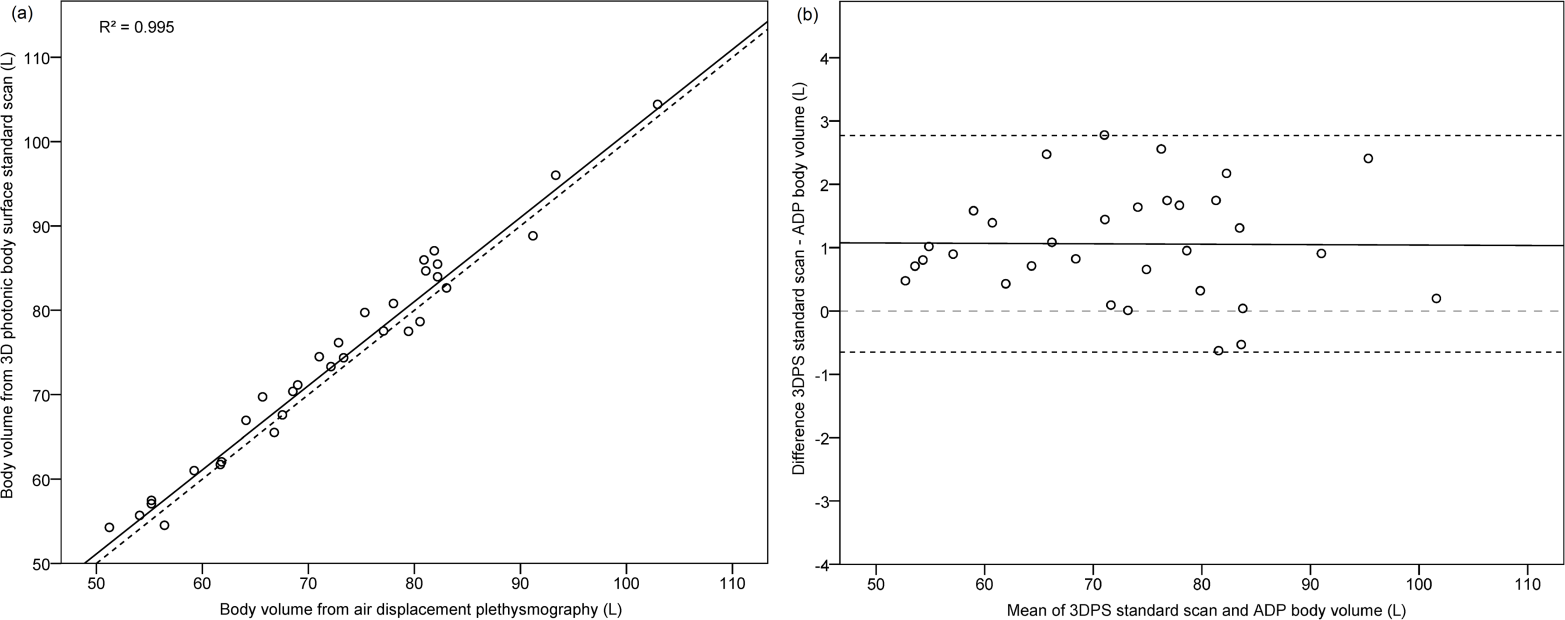
Body volume measurement agreement between 3D photonic body surface standard scan and air-displacement plethysmography (N = 32). (a) Body volume measured by 3DPS and ADP plotted on the regression line. Dashed line: line of identity (y = 1*x + 0). (b) Differences in body volume measurements from 3DPS and ADP plotted against their mean. Solid line: mean measurement difference (3DPS–ADP), dashed lines: limits of agreement.

Fig 1A and 1B illustrate the agreement of BV from 3DPS StdScan and ADP based on correlation analyses and Bland-Altman-Plots. Both measures were strongly correlated (R = 0.99, Fig 1A, Table 2). On average, ADP BV was 72.2 ± 12.4 L; the 3DPS BVs were slightly higher by 1.1 ± 0.9 L (p<0.001), 1.0 ± 0.8 L (p<0.001), and 2.5 ± 1.0 L (p<0.001), in standard, relaxed and exhaled position, respectively (Table 2). There was no significant correlation of the measurement difference between the two methods with their mean (Table 2). When stratified by sex, BMI or age, the differences between 3DPS and ADP volume measurements were slightly, albeit not significantly, higher in men (1.3 ± 1.0 L) than women (0.8 ± 0.7 L, p = 0.09), in non-overweight (1.2 ± 0.8 L) compared to overweight individuals (0.8 ± 1.0 L, p = 0.23) and in participants aged <30 years (1.2 ± 1.0 L) compared to >30 years (1.0 ± 0.8 L, p = 0.60).

**Table 2:** Body volume and %body fat from 3D photonic body surface scans (3DPS) and air-displacement plethysmography (ADP) and measurement differences between both methods.

ADP			3DPS				difference 3DPS—ADP				correlation 3DPS with ADP	correlation difference (3DPS-ADP) with mean
mean	SD	95% CI	scan type	mean	SD	95% CI	mean	SD	95% CI	p	R	R
**body volume (L)**												
72.2	12.4	(67.8–76.7)	standard	73.3	12.4	(68.8; 77.7)	1.1	0.9	(0.7;1.4)	< .001	0.998	-0.010
			relaxed	73.2	12.3	(68.8; 77.6)	1.0	0.8	(0.7;1.3)	< .001	0.998	-0.088
			exhaled	74.7	12.5	(70.2; 79.2)	2.5	1.0	(2.2;2.9)	< .001	0.997	0.097
**%body fat (%)**^**a**^												
23.7	11.6	(19.4–27.9)	standard	30.7	9.3	(27.3; 34.1)	7.0	5.6	(5.0;9.1)	< .001	0.893	-0.431
			relaxed	30.3	9.1	(26.9; 33.6)	6.6	5.3	(4.7;8.5)	< .001	0.909	-0.488
			exhaled	40.2	8.8	(37.0; 43.5)	16.6	6.5	(14.2;19.0)	< .001	0.848	-0.481

^a^ 1 case was excluded due to invalid %body fat.

**Fig 2:**
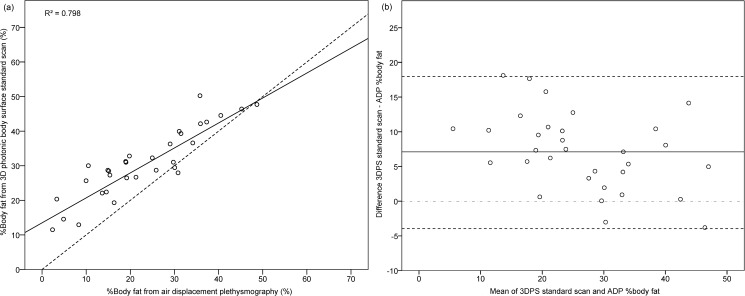
%Body fat measurement differences between 3D photonic body surface standard scan and air-displacement plethysmography (N = 31). (a) %Body fat estimated from 3DPS and ADP plotted on the regression line. Dashed line: line of identity (y = 1*x + 0). (b) Differences in %body fat measurements from 3DPS and ADP plotted against their mean. Solid line: mean measurement difference (3DPS–ADP), dashed lines: limits of agreement.

For the comparison of %BF, one participant was excluded from the analysis due to invalidly low %BF (<1% BF) given by ADP. %BF based on 3DPS StdScan and on ADP were highly correlated (R = 0.89, Fig 2A, Table 2), but %BF based on 3DPS was on average higher by 7.0 ± 5.6% (p<0.001), 6.6 ± 5.3% (p<0.001) and 16.6 ± 6.5% (p<0.001) for StdScan, RlxScan, and ExhScan (Table 2), respectively, as compared to a mean %BF from ADP of 23.7 ± 11.6%. There was a moderate inverse correlation of the difference in %BF between 3DPS and ADP with their mean (Table 2), indicating that with increasing body fat, the %BF difference between ADP and 3DPS decreased (Table 2, Fig 2B). The difference between 3DPS StdScan and ADP was again non-significantly higher in men (8.2 ± 6.4%) compared to women (5.8 ± 4.7%, p = 0.25), higher in non-overweight (8.7 ± 5.0%) compared to overweight individuals (5.0 ± 5.8%, p = 0.08), and higher in persons <30 years (8.5 ± 7:0%) compared to >30 years (6.6 ± 5.2%, p = 0.50).

Regression analysis revealed no significant contribution of gender, age, BMI, or the interaction of BV*gender for the prediction of ADP BV from 3DPS StdScan BV (Table 3). After stepwise exclusion, the equation for calibration of the StdScan BV was:

$$StdScan\;BV_{calibrated}=0.998\times StdScan\;BV-0.928$$ (7)

**Table 3:** Linear regression models for the prediction of air-displacement plethysmography (ADP) measured body volume and %body fat on basis of 3D photonic body surface (3DPS) standard scan.

	regression coefficient^a^	95% CI	standardized coefficient	p	R^2^
1a: Full model for the prediction of ADP body volume from 3DPS standard scan body volume (in L)					
intercept	-4.522	(-8.381; 0.663)		0.023	0.995
standard scan body volume	1.006	(0.930; 1.081)	1.005	< .001	
BMI (kg/m^2^)	0.122	(-0.078; 0.323)	0.042	0.219	
gender (male = 0, female = 1)	4.364	(-0.340; 9.067)	0.179	0.068	
age (years)	-0.159	(-0.688; 0.369)	-0.008	0.540	
gender*body volume	-0.056	(-0.119; 0.008)	-0.164	0.083	
1b: Final model for the prediction of ADP body volume from 3DPS standard scan body volume (in L)					
intercept	-0.928	(-2.881; 1.026)		0.340	0.995
standard scan body volume	0.998	(0.972; 1.024)	0.998	< .001	
2a: Full model for the prediction of ADP %body fat from 3DPS standard scan %body fat (in %)					
intercept	-29.979	(-50.530; 9.427)		0.006	0.806
standard scan %body fat	0.854	(0.462; 1.247)	0.690	< .001	
BMI (kg/m^2^)	1.054	(0.124; 1.984)	0.368	0.028	
gender (male = 0, female = 1)	22.246	(-3.458; 47.951)	0.934	0.087	
age (years)	-0.726	(-4.073; 2.621)	-0.039	0.659	
gender*%body fat	-0.266	(-0.649; 0.117)	-0.804	0.165	
2b: Final model for the prediction of ADP %body fat from 3DPS standard scan %body fat (in %)					
intercept	-10.284	(-16.823; 3.744)		0.003	0.791
standard scan %body fat	1.106	(0.898; 1.313)	0.893	< .001	

^a^For the intercept the regression coefficient can be interpreted as a constant that is added to the regression (prediction) model. For the remaining variables, the regression coefficient can be interpreted as the change in BV or the change in %BF per 1-unit change in the variables listed in the table.

Similar results were found for the regression of ADP %BF from 3DPS StdScan %BF (Table 3) with the following calibration equation after stepwise exclusion of aforementioned potential contributors:

$$StdScan\;\%BF_{calibrated}=1.106\times StdScan\;\%BF-10.284$$ (8)

### Reliability of 3DPS

To assess reliability, the 3DPS measurement was repeated after four weeks. Reliability was high for BV with a mean difference of 0.1 ± 1.3 L, 0.2 ± 1.3 L, and 0.2 **±** 1.2 L for StdScan, RlxScan, and ExhScan, respectively, and ICCs of >0.998 (Table 4). %BF based on StdScan had a mean difference of -0.4 ± 2.4% and ICCs ranged from 0.945 to 0.983 (Table 4). The differences between both 3DPS measurements were not statistically significantly different from zero.

**Table 4:** Reliability of the 3D photonic body surface scanner.

scan type	scan 1		scan 2		difference scan 1–2			reliability	
	mean	SD	mean	SD	mean	SD	p	ICC	95% CI
**body volume (L)**									
standard	71.7	16.0	71.6	15.5	0.1	1.3	0.75	0.998	(0.996; 0.999)
relaxed	71.7	16.0	71.5	15.3	0.2	1.3	0.52	0.998	(0.996; 0.999)
exhaled	73.2	16.1	73.0	15.6	0.2	1.2	0.49	0.999	(0.996; 0.999)
**%body fat**									
standard	33.2	8.9	33.6	8.5	-0.4	2.4	0.49	0.982	(0.952; 0.993)
relaxed	32.8	8.9	32.6	8.6	0.2	2.3	0.72	0.983	(0.955; 0.994)
exhaled	43.2	7.9	42.9	7.2	0.3	3.5	0.72	0.945	(0.854; 0.980)

## Discussion

The aim of our study was to investigate validity and longer-term reliability of 3DPS based BV and %BF over an approximately four weeks period for an application in epidemiological studies in the general adult population. Our study evaluating the validity of BV and %BF measurements from 3DPS in comparison to measurements of APD showed a strong correlation of the results from both methods, although our data also indicated that measurements derived by 3DPS were on average higher than those derived by ADP. Further, we found high reliability in the measurement of BV and %BF using 3DPS over a period of four weeks. Of the three 3DPS scans that we used, the scan in relaxed position showed the strongest agreement to ADP.

The measurement differences in BV found in our study are somewhat similar to what was found in other validation studies for other 3DPS scanners using ADP or underwater weighing (UWW) as reference methods. A study from Wells et al. with 22 adults using a Hamamatsu Bodyline Scanner reported an underestimation of 0.3 ± 2.7 L against ADP and overestimation of 0.5 ± 2.4 L for UWW, respectively [30]. Similarly, Wang et al. revealed an overestimation of 0.5 ± 0.1 L compared with UWW for an updated version of the Hamamatsu scanner in adult men and women [31], whereas Pepper et al. found a mean difference of 0.2 L in middle-aged women using a rotary laser scanning system developed by their research group [32]. In contrast, analyses from Ng et al. with a Fit3D Proscanner resulted in a 4.2 L (95% CI: −5.13; −3.17) smaller BV measured by 3DPS compared to ADP [33]. Differences in the BV measurement agreement between 3DPS and ADP among the different studies may have several reasons. First, various body surface scanner models from different manufacturers were employed in these studies, which vary regarding point density, scan time, the number of cameras and laser used, and hardware components. Further, the scanning procedure proposed by the manufacturer differed between studies. Additionally, the scanner software is proprietary to the manufacturers and it remains unclear how the algorithm of a specific scanner model calculates BV from the point cloud. Further, varying study populations could have contributed to differences in the measurement agreement reported in the studies. Finally, different approaches for the adjustment of BV based on 3DPS scans for lung volume were used. Lung volume was either predicted from weight and height similarly to our approach [30], was measured by spirometry [31], or no adjustment was made [32]. Thus, our results are not in accordance with the study of Wang et al., which used spirometry to directly measure residual volume and subsequently correct a scan after maximum expiration [31], since we found the highest discrepancy from ADP for the scan after maximum expiration corrected for predicted residual volume. However, spirometry measurements probably give more valid estimates than the equation used in our analysis. Moreover, in spirometry, the participants are instructed to breathe out as much as possible necessarily with some effort. However, this was not practical during the 3DPS examination, since it would have influenced the posture of the participants. Therefore some expiratory reserve volume was probably retained in the lungs and accounted, in part, for the high discrepancy of the 3DPS exhaled scan compared to ADP, if only the FRC was deducted from 3DPS.

Taken together, the calculation rather than measurement of TGV (or residual volume) could have resulted in discrepancies from actual TGV values on an individual level, for which we could not account for. Though this similarly affected both ADP and 3DPS, the ongoing approaches to adjust for TGV differed between methods, e.g. ADP BV was adjusted for 40% of TGV, whereas it was completely deducted from 3DPS BV.

The 3DPS does not follow the standardized WHO measurement protocol for assessment of height. In this regard, we previously showed that the VitusSmart XXL 3DPS overestimates body height by on average 0.9 cm compared to manual measurements on basis of standardized WHO guidelines [13]. However, this is unlikely to account for the differences in the measurement of BV between 3DPS and ADP since we used manual height measurements following the WHO protocol to calculate TGV in our analyses.

Furthermore, other factors independent of the scanner’s ability to validly estimate BV could have contributed to the overestimation of BV. 3DPS visually scans the body surface and thus, any body-enlarging object, like clothing and hair, amplifies BV. Even though this influence was minimized by wearing tight-fitting underwear and a bathing cap, it could not be completely eliminated, e.g. if the participant had a large hair volume, a beard, thick body hair, or padded underwear. In addition, participants may have had the tendency to hold their breath or breathe flatly during the scanning procedure in order to not blur the picture, which could have led to a higher air volume in their lungs.

While we observed an acceptable difference between 3DPS and ADP for BV, the agreement between methods found for %BF was lower. A non-zero intercept and non-identity slope in the Bland Altman plots reflected the estimation of %BF on the basis of 3DPS BV to yield higher values than estimation based on ADP. Similar graphical findings were also reported in a study by Wang et al. using underwater weighing as comparison method to evaluate validity of 3DPS based BV and %BF [34]. Bearing in mind that the regression lines of BV and %BF are independent from each other, these differences may mainly be due to the fact that there is no linear association between BV and %BF. This is attributable to the formulas used to derive %BF from BV [23, 24], which include body density as ratio of body mass to BV, with BV being indirectly assessed using 3DPS or ADP in our study (Eqs 2 and 3) [23, 24]. As a consequence, small deviations in BV between 3DPS and ADP might result in proportionally pronounced deviations in %BF. This sensitivity of Siri’s and Brozek’s formula to small differences in BV estimation is well known [35]. In our study, we found a mean BV difference of 1.1 L for the StdScan to result in a 7.0% higher mean %BF. Wells et al. found differences in %BF between 3DPS and ADP of -2.6 ± 21.4% using a Hamamatsu Bodyline scanner [30] and Wang et al. found differences in %BF of 0.7 ± 1.0% when another Hamamatsu scanner was used [31]. Pepper et al. yielded a 1.4% higher %BF for 3DPS than for ADP, but a 1.9% lower estimate against dual X-ray absorptiometry [32]. In consequence, this could lead to misclassification regarding the allocation to normal or elevated %BF. In our study, 39% of the participants were differentially allocated to normal vs. elevated %BF on the basis of ADP and 3DPS, meaning that they had normal %BF based on ADP but elevated %BF based on 3DPS [36].

Furthermore, in contrast to BV, for %BF we found a correlation between the difference and the mean of 3DPS and ADP measurements, with higher estimates based on 3DPS than for ADP observed for lower %BF, while with increasing %BF the opposite was true. A similar dependency of the difference between methods and the mean %BF was also found by Wang et al. [34]. This skew in the comparison might be due to the fact that for persons with a BMI of <18.5 kg/m^2^ (as well as for persons with BMI >30.0 kg/m^2^) Brozek’s [24] instead of Siri’s formula is used to derive %BF from BV [23]; using two different formulas might to some extent distort the comparability along the entire %BF spectrum. However, the differences in %BF between 3DPS and ADP were within the limits of agreement according to the Bland Altman plots.

In respect to the reliability of the 3DPS, it should be mentioned that a single assessment of a biomarker, as has often been done in baseline examinations of large-scale epidemiological studies, may be susceptible to short-term variation and not reflect true long-term exposure. Therefore, random measurement error generally tends to decrease correlation and regression coefficients in epidemiological studies toward 0 and bias relative risks toward 1. However, in our analysis, the 3DPS scanner revealed an excellent reliability after a time period of four weeks. To our knowledge, reliability over a longer-time period has not been analyzed previously; however, other studies with consecutive measurements showed very good short-term reliability [13, 30–32]. These findings suggest that a single measurement of BV or %BF may be sufficient for risk assessment in epidemiological studies. Furthermore, as a low reliability for a risk factor measurement tend to attenuate exposure-disease associations, ICCs derived from our study may also enable to deattenuate estimates of observed relative risks to approximate true relative risks in cohort studies [15].

Our study has some strengths and limitations. ADP has been shown to be an accurate method for assessment of BV, and it is probably the method that is most often used in experimental studies [37–39]. However, ADP is based on the two-compartment model of body composition estimation, and it presumes a constant density of fat free mass, which is not always appropriate, e.g. in old age or in athletes [40]. Hence, other methods like dual X-ray absorptiometry or magnetic resonance imaging could give more valid %BF estimates and thus might be the preferred ‘gold standard’ for 3DPS validation. The sample sizes of our validity and reliability study were relatively small and did not aim to be representative of the general population; however, the distribution of characteristics of study participants was broad, and, therefore, our findings should be applicable to populations with similar characteristics. Further, we found narrow confidence intervals for validity with most p-values being highly significant; thus, our sample sizes, although small, were adequate to produce conclusive results. Nevertheless, validity and reliability in subjects with different phenotypes (e.g., diseased populations) need to be investigated in future studies.

In conclusion, our study showed good agreement between 3DPS and ADP for measurement of BV and %BF, although measurements were slightly higher for 3DPS compared to ADP. Further, we found high ICCs for BV and %BF when measured with 3DPS four weeks apart. These data indicate good validity and excellent four-week reliability for an application of 3DPS to assess BV and %BF in an epidemiological study.
